# Development of Bio-Based Benzoxazine V-fa/PEG/Carbon Black Composites: Thermal and Mechanical Properties

**DOI:** 10.3390/polym17202776

**Published:** 2025-10-16

**Authors:** Nattapon Chaiwichian, Chaitawat Saelee, Kamontip Kuttiyawong, Sarawut Rimdusit, Kasinee Hemvichian, Pattra Lertsarawut, Sunan Tiptipakorn

**Affiliations:** 1 Department of Physical and Material Sciences, Faculty of Liberal Arts and Science, Kasetsart University, Nakhon Pathom 73140, Thailand; nattapon.chaiw@ku.th (N.C.); chaitawat.sa@ku.th (C.S.); faaskmt@ku.ac.th (K.K.); 2 Center of Excellence in Polymeric Materials for Medical Practice Devices, Department of Chemical Engineering, Faculty of Engineering, Chulalongkorn University, Bangkok 10330, Thailand; sarwut.r@chula.ac.th; 3 Thailand Institute of Nuclear Technology (Public Organization), Ongkarak District, Nakornnayok 26120, Thailand; kasinee@tint.or.th (K.H.); pattra@tint.or.th (P.L.)

**Keywords:** bio-based polybenzoxazine, polyethylene glycol, different molecular weights, carbon black, composites

## Abstract

In this study, the blends of bio-based polybenzoxazine (V-fa type) and poly(ethylene glycol) (PEG) with PEG contents from 50 to 95 wt% and different molecular weights were developed to improve the flexibility of thermosetting polymers. Of these blends, PEG 8k at 80 wt%, which exhibited the best processability, was selected for further development via compositing with carbon black (CB) from 0 to 20 phr. Differential Scanning Calorimetry (DSC) analysis revealed that the melting temperature (T_m_) increased from 70 to 83 °C and glass transition temperatures (T_g_) increased from –53 to –48 °C at 20 phr. Thermogravimetric Analysis (TGA) demonstrated high thermal stability, with T_dmax_ (for all CB contents) presented at ca. 416 °C. Moreover, char yield was increased from 10% (without CB) to 28% (20 phr), reflecting improved decomposition resistance. Mechanical properties demonstrated that CB significantly reinforced the composites. The flexural modulus and flexural strength were increased from 117.18 MPa (without CB) to 456 MPa (10 phr) and from 2.42 MPa (without CB) to 3.94 MPa (2.5 phr), respectively. The SEM images confirmed uniform morphology and good filler dispersion. Conclusively, the composites of 8k PEG 80 wt% filled with 2.5 phr of CB provided an optimal balance of mechanical and thermal stability and engineering polymer applications.

## 1. Introduction

Polymers derived from renewable resources have gained increasing attention in recent years, driven by environmental concerns and the growing demand for sustainable materials. Compared to petroleum-based polymers, bio-based polymers provide significant environmental benefits, such as reduced greenhouse gas emissions, decreased dependence on fossil fuels, and utilization of renewable raw materials [1]. In addition, bio-based polymers are highly versatile, and their chemical structure can be tailored to achieve desirable thermal, mechanical, and chemical properties, making them suitable for a wide range of industrial and technological applications.

Among various bio-based polymers, bio-based benzoxazines have emerged as a particularly attractive class of thermosetting polymers. They combine the inherent advantages of benzoxazine chemistry, including excellent thermal stability, low curing shrinkage, and superior chemical resistance, with the sustainability offered by renewable phenolic sources [2]. Recent studies have demonstrated the potential of bio-based benzoxazine composites in structural, electronic, and environmentally friendly applications, highlighting their competitiveness with traditional petroleum-based thermosets. For example, fully bio-based benzoxazine composites reinforced with natural fibers or bio-derived fillers have been reported to achieve enhanced mechanical strength and improved thermal stability [3], while hybrid bio-benzoxazine systems incorporating inorganic nanofillers have shown multifunctional properties suitable for advanced structural and electronic applications [4].

Nevertheless, despite these advantages, a notable limitation of polybenzoxazine, particularly V-fa, is its inherent brittleness and rigidity. Consequently, this restricts their applicability in systems that require toughness, flexibility, or dynamic mechanical behavior unless the polymer network is modified with softer or more elastic components. To overcome these drawbacks, several strategies have been employed, such as copolymerization with flexible segments, blending with other thermosets, and incorporation of fillers or nanofillers to enhance the strength and reliability [5]. For example, incorporating flexible bio-based segments like eugenol-derived benzoxazines (E-fa) has demonstrated favorable thermal and mechanical properties, providing a foundation for the development of sustainable high-performance polymer systems, and blending with epoxy resins has been shown to improve ductility and processability [6]. Smart material design using fillers has also attracted attention, introducing shape-memory, self-healing, and responsive behaviors into benzoxazine-based composites [7].

Within this context, V-fa, a novel benzoxazine monomer synthesized from vanillin and furfurylamine, offers a sustainable route to functional PBZs. Its unique aromatic structure provides high thermal stability and network rigidity [2]. To further improve flexibility and processability, poly(ethylene glycol) (PEG) was incorporated into the polymer system. PEG was a widely used, biocompatible, and water-soluble polymer known for its non-toxicity and chain flexibility. PEG makes it ideal for plasticizing rigid networks and promoting hydrogen-bonding interactions with poly(V-fa) hydroxyl groups. The inclusion of PEG into rigid polybenzoxazine matrices can enhance chain mobility, reduce brittleness, and improve both mechanical performance and processability. Its compatibility with polybenzoxazine makes it an ideal soft segment for creating polymer blends with enhanced thermal, mechanical, and morphological properties [8].

In addition to PEG, the introduction of fillers into polymer systems is an effective strategy to further enhance strength, thermal stability, and multifunctionality. Various inorganic fillers such as silica, nano clay, graphene oxide, and carbon nanotubes have been incorporated into benzoxazine or thermosetting resins to improve modulus, strength, or conductivity [5,7]. Among these, CB was selected as the reinforcing filler for V-fa/PEG blends in this study due to its unique advantages. CB provides excellent dispersion, high surface area, strong interfacial interactions with polymer chains, and the ability to enhance energy dissipation under stress. Unlike rigid nanoplatelets or fibers, CB can reinforce the matrix without excessively compromising flexibility, offering a distinctive balance of stiffness, toughness, and multifunctional performance. Moreover, CB is cost-effective, widely available, and industrially scalable, yet its application in bio-based V-fa/PEG systems has not been previously explored [9,10].

Therefore, the present study focuses on the systematic investigation of how variations in PEG content and molecular weight affect blend homogeneity and flexibility and on the synthesis of V-fa benzoxazine/PEG polymer blends and their reinforcement with CB filler to produce advanced composites with improved thermal, mechanical, and morphological properties of the resulting materials. The chemical structures were characterized using Fourier Transform Infrared Spectroscopy (FT-IR), thermal behaviors were assessed by Thermogravimetric Analysis (TGA) and Differential Scanning Calorimetry (DSC), and mechanical properties were evaluated using a universal testing machine. In addition, morphology was examined using Scanning Electron Microscopy (SEM). The novelty of this work lies in the unique combination of V-fa/PEG with CB, which has not been previously reported. By leveraging the synergistic effects of PEG-induced flexibility and CB-induced reinforcement, the study not only mitigates the inherent brittleness of poly(V-fa) but also produces composites with enhanced strength, durability, and multifunctionality. The findings of this research provided fundamental insights into the design of environmentally friendly polymer systems with optimized flexibility and processability. These materials show potential for applications in functional performance and sustainability of materials and are feasible to be further developed as shape-memory polymer systems in the future.

## 2. Materials and Methods

### 2.1. Chemicals and Materials

Bio-based benzoxazine resin (V-fa type) was synthesized from vanillin, furfurylamine, and paraformaldehyde. Vanillin (C_8_H_8_O_3_, ReagentPlus^®^ grade, 99% purity) was purchased from Sigma-Aldrich Co., Ltd. (St. Louis, MO, USA). Furfurylamine (C_5_H_7_NO, 99% purity) was obtained from Sigma-Aldrich Co., Ltd. (St. Louis, MO, USA). Paraformaldehyde ((CH_2_O)_n_, for synthesis) was supplied by Merck KGaA (Darmstadt, Germany). Polyethylene glycol (PEG, H(OCH_2_CH_2_)_n_OH) with molecular weights of 4000, 8000, and 20,000 Da was also purchased from Sigma-Aldrich Co., Ltd. (St. Louis, MO, USA). Carbon black (ENSACO 250G).

### 2.2. Preparation of V-fa/PEG Blends

**Figure 1:**
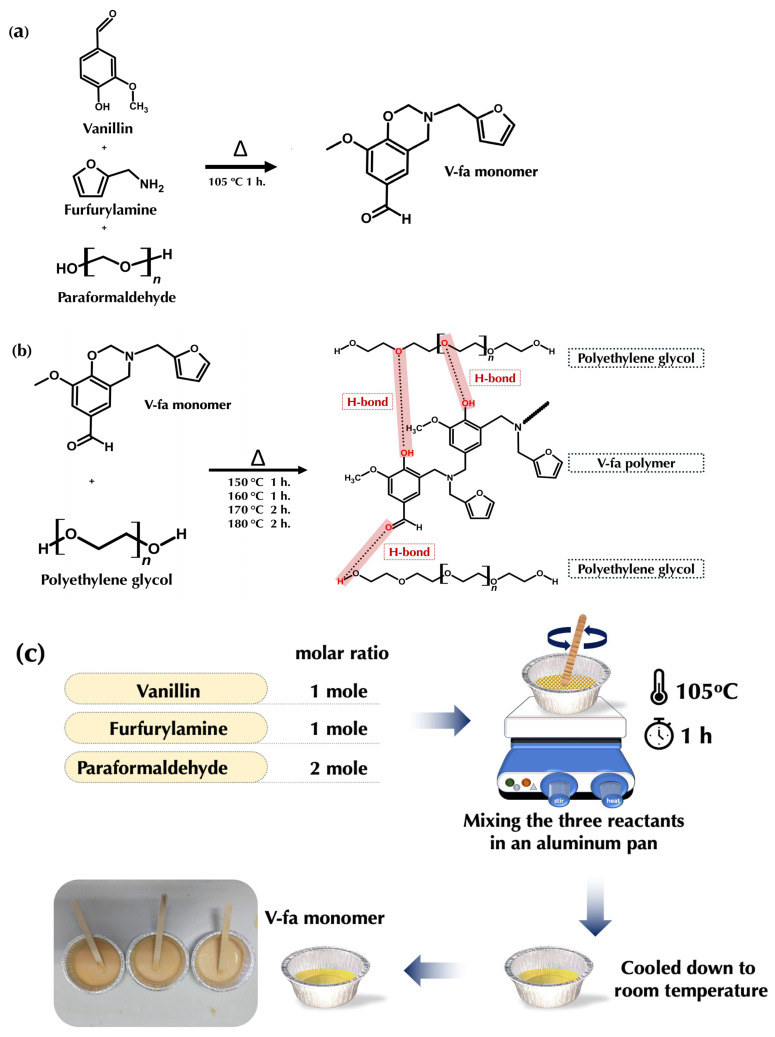

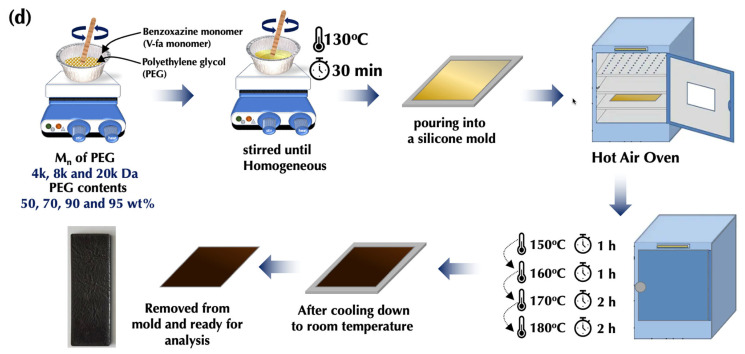
Synthesis of vanillin-based benzoxazine: (**a**) structure of synthesized V-fa monomer; (**b**) structure of synthesized V-fa/PEG polymer blends; (**c**) preparation of V-fa monomer; (**d**) preparation of V-fa/PEG polymer blends.

The bio-based V-fa benzoxazine monomer was synthesized by mixing vanillin, furfurylamine, and paraformaldehyde (at a molar ratio of 1:1:2), which were homogeneously mixed according to the solvent-free synthesis method shown in Figure 1c [11]. All mixed components were heated at 105◦C for 60 min. After cooling down the mixtures, the resulting yellow monomer was obtained in solid form. The synthesis route of the bio-based V-fa benzoxazine monomer is illustrated in Figure 1a. The bio-based benzoxazine monomer was mixed with polyethylene glycol, molecular weights of 4000, 8000, and 20,000 Da, using PEG contents of 50, 70, 80, 90, and 95 wt%. The mixtures were prepared in aluminum foil containers. Each mixture was then heated and stirred using a hotplate at 130 °C for 30 min until a homogeneous blend was obtained. The homogeneous mixture was poured into silicone molds and subsequently cured in a hot air oven at 150 °C and 160 °C for 1 h each, followed by curing at 170 °C and 180 °C for 2 h each shown in Figure 1d [2]. The polymerization and crosslinking process of the V-fa/PEG blends is schematically shown in Figure 1b. After curing, the samples were allowed to cool to room temperature and were then removed from the mold.

### 2.3. Preparation of V-fa/PEG/CB Composites

The composites were prepared by incorporating CB into the V-fa/PEG blends. The bio-based V-fa benzoxazine monomer was first synthesized as described in Section 2.2. The desired PEG (molecular weights of 4000, 8000, and 20,000 Da) was then added to the monomer at PEG contents of 50, 70, 80, 90, and 95 wt%. CB was incorporated in the range from 0 to 20 phr based on the total weight of the V-fa/PEG blends. The monomer, PEG, and CB were thoroughly mixed in aluminum foil containers using a mechanical stirrer and heated on a hot plate at 130 °C for 30 min to obtain a homogeneous mixture. The mixture was then poured into silicone molds and cured in a hot air oven using the same temperature profile as the blends: 150 °C and 160 °C for 1 h each, followed by 170 °C and 180 °C for 2 h each [2]. After curing, the composites were cooled to room temperature and demolded for subsequent characterization.

### 2.4. Characterizations

The structural characterizations of the V-fa monomer and the corresponding poly(V-fa) polymer were investigated using both Fourier Transform Infrared (FTIR) spectroscopy and solid-state ^13^C Nuclear Magnetic Resonance (NMR) spectroscopy. The FTIR spectra were recorded with a Fourier Transform Infrared Spectrometer (Bruker, model Tensor 27, Mannheim, Germany) equipped with a ZnSe crystal in attenuated total reflectance (ATR) mode, using a resolution of 4 cm^−1^ and 32 scans per sample. This technique was employed to analyze the functional groups of pure PEG with different molecular weights (4k, 8k, and 20k), V-fa monomer, poly(V-fa), and blend polymers at various weight ratios, as well as composites containing different contents of CB.

To further confirm the chemical structure and polymerization of the V-fa monomer, solid-state ^13^C NMR spectroscopy was performed using a Fourier Transform Nuclear Magnetic Resonance Spectrometer (Bruker, model Avance III HD/Ascend 400 WB, Mannheim, Germany) operating at 400 MHz. The spectra of V-fa monomer, poly(V-fa), and PEG with different molecular weights were obtained and compared. This analysis provided direct evidence of the disappearance of the characteristic oxazine ring carbons in the monomer upon polymerization, as well as the presence of new carbon signals associated with the poly(V-fa) network structure. The combined FTIR and ^13^C NMR results were used to confirm the chemical structures of the monomer, polymer, blends, and composites and to investigate the specific interactions between PEG, V-fa, and CB.

The thermal properties of the V-fa/PEG blends were investigated using a Differential Scanning Calorimeter (DSC, METTLER TOLEDO, model DSC822e, Greifensee, Zürich, Switzerland). Approximately 3–5 mg of each blend sample was sealed in an aluminum DSC pan and heated from −100 to 270 °C at a heating rate of 20 °C/min under a nitrogen flow of 60 mL/min. The melting temperature (T_m_) and enthalpy of fusion (∆H) were determined from the second heating cycle. These parameters were used to compare the influence of PEG molecular weight and blend ratio on the crystallization behavior of the blends.

The curing behavior of the V-fa monomer was also examined using DSC to determine the optimal curing conditions for converting V-fa monomer into poly(V-fa). Approximately 3 mg of the V-fa monomer was placed in an aluminum DSC pan and heated from −100 to 260 °C at a heating rate of 20 °C/min under a nitrogen flow of 60 mL/min. The curing process was evaluated at different stepwise curing schedules, and the degree of conversion (%Conversion) was calculated according to Equation (1), following the procedure reported by Rimdusit et al. (2009) [12].

(1) $$\%\mathrm{Conversion}=\left[1-\frac{H_{\mathrm{rxn}}}{H_{0}}\right]\times \;100$$

where H_rxn_ is the heat of polymerization of the V-fa monomer under the studied curing condition, and H_0_ is the total heat of polymerization for the uncured V-fa monomer. This analysis allowed the identification of suitable thermal curing conditions for achieving high conversion of V-fa monomer to poly(V-fa).

Thermal stability was analyzed using a Thermogravimetric Analyzer (TGA, METTLER TOLEDO, model TGA/DSC 2 STARe System, Greifensee, Zürich, Switzerland). About 10 mg of each sample was heated from 40 to 800 °C at a heating rate of 20 °C/min under a nitrogen atmosphere under a nitrogen flow of 60 mL/min, and the maximum decomposition temperature (T_d,max_) as well as the char yield at 800 °C were recorded.

Flexural properties of the CB-reinforced composites were evaluated using a Universal Testing Machine (Instron^®^, Norwood, MA, USA) in accordance with ASTM D790 [13]. Rectangular specimens with dimensions of 20 × 80 × 2 mm^3^ were tested under three-point bending mode. The flexural strength and modulus were calculated from the load–load-displacement data to assess the influence of CB on the mechanical performance of the composites.

The fracture surfaces of the flexurally tested specimens were examined using a Scanning Electron Microscope (SEM, JEOL, model JSM-IT500HR, JEOL Ltd., Akishima, Tokyo, Japan). Prior to observation, samples were sputter-coated with gold to minimize charging effects. Fracture morphologies were captured at magnifications of 1000×. Composites with different CB contents were analyzed to evaluate crack propagation, particle dispersion, and interfacial adhesion within the polymer matrix.

The water solubility behavior of the polymer blends was assessed qualitatively by immersing rectangular specimens (20 × 20 × 2 mm^3^) in deionized water at room temperature. The dissolution and interaction of PEG with water were observed over time.

## 3. Results and Discussion

### 3.1. Part I—Preparation of V-fa/PEG Polymer Blends

#### 3.1.1. Synthesis and Characterizations of Bio-Based V-fa Benzoxazine

**Figure 2:**
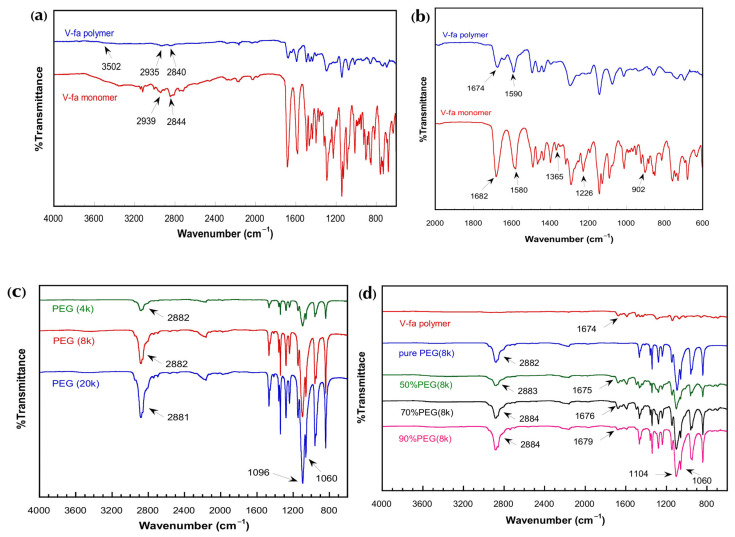
FT-IR spectra: (**a**) V-fa monomer and V-fa polymer in the range of 600–4000 cm^−1^; (**b**) V-fa polymer in the range of 600–2000 cm^−1^; (**c**) FT-IR spectra of PEG with different molecular weights; (**d**) V-fa polymer/PEG blends with PEG molecular weight of 8000 Da at 50–90 wt%.

**Figure 3:**
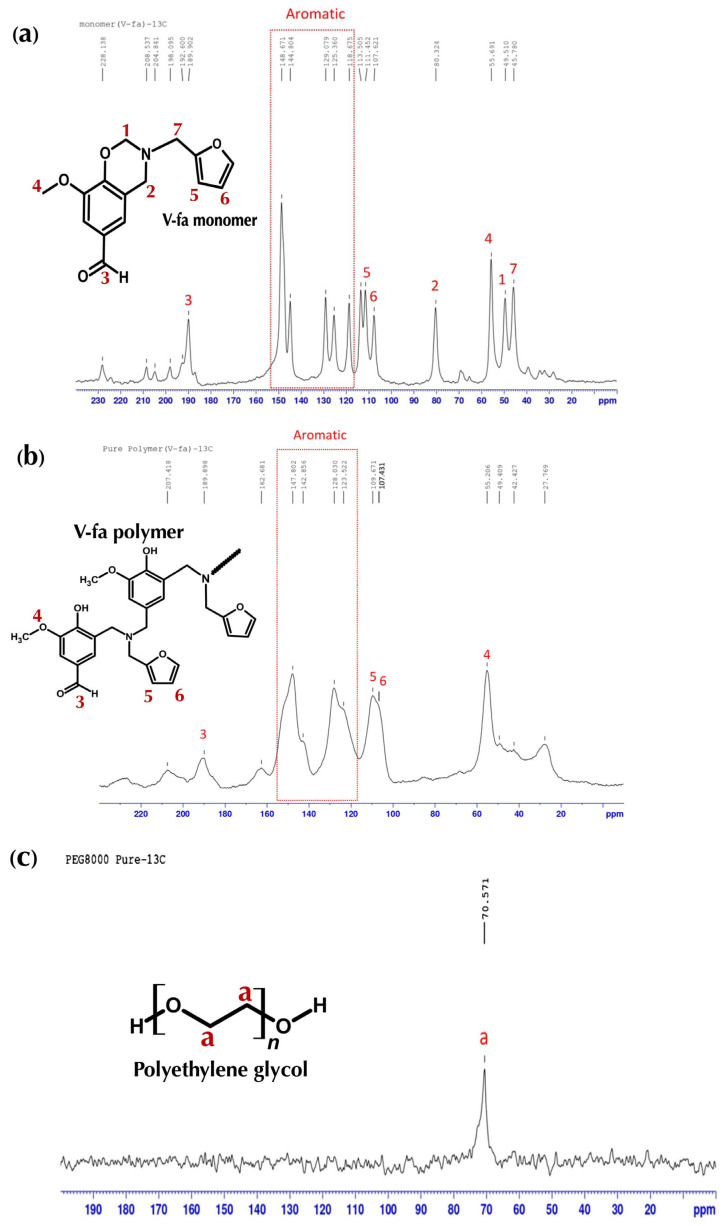
Solid-state ^13^C NMR spectra: (**a**) V-fa monomer; (**b**) V-fa polymer; (**c**) PEG.

The bio-based V-fa benzoxazine was successfully synthesized via a solvent-free Manniche condensation of vanillin, furfurylamine, and paraformaldehyde at a molar ratio of 1:1:2, following standard procedures for bio-based benzoxazines. In this reaction, the phenolic hydroxyl group of vanillin reacted with the primary amine of furfurylamine in the presence of formaldehyde, leading to the formation of the oxazine ring through nucleophilic attack of the phenolic oxygen on the electrophilic methylene carbon adjacent to the amine. Upon heating at 105 °C for 60 min, a yellow solid monomer was obtained with an isolated yield of approximately 85–88%, indicating high efficiency of the solvent-free synthesis method [14,15]. The structure of V-fa was confirmed by FTIR and solid-state ^13^C NMR analyses. The FTIR spectra of V-fa monomer and polymer are shown in Figure 2a,b, and the corresponding solid-state ^13^C NMR spectra are presented in Figure 3a,b. FTIR spectra showed CH_2_ asymmetric and symmetric stretching at 2939 and 2844 cm^−1^, carbonyl (C=O) peaks at 1682 and 1674 cm^−1^, furan ring peaks at 1580 and 1590 cm^−1^, a tetra-substituted benzene peak at 1365 cm^−1^, and oxazine ring vibrations at 902 and 1226 cm^−1^. In the ^13^C NMR spectrum, the oxazine ring carbons (CH_2_N and CH_2_O) appeared at 49.510 and 80.324 ppm, aromatic carbons from 118.675 to 148.671 ppm, the methyl carbon of furan at 55.691 ppm, the furan ring carbons at 109.671–111.452 ppm and 107.621 ppm, and the CH_2_ carbons attached to nitrogen at 45.780 ppm [16].

#### 3.1.2. Synthesis and Characterizations of the Polymer Blends Between Bio-Based V-fa Benzoxazine and PEG

Thermal polymerization of V-fa into poly(V-fa) was achieved by curing the monomer in the presence of PEG with molecular weights of 4000, 8000, and 20,000 Da at various PEG contents (50–95 wt%). The corresponding FTIR spectra of PEG with different molecular weights and V-fa/PEG blends are illustrated in Figure 2c,d. The FTIR spectra of V-fa polymer showed slight shifts in CH_2_ stretching to 2935 and 2840 cm^−1^, disappearance of oxazine ring peaks at 902 and 1226 cm^−1^, and the appearance of a broad −OH stretching peak at 3502 cm^−1^, confirming ring-opening polymerization and formation of phenolic hydroxyl groups. In solid-state ^13^C NMR spectra (Figure 3a–c), distinct chemical shifts further confirm successful polymerization and PEG incorporation. The spectrum of the V-fa monomer (Figure 3a) displays characteristic oxazine-related carbon peaks that completely disappear after polymerization. The poly(V-fa) spectrum (Figure 3b) exhibits aromatic carbons shifted to 123.522–147.802 ppm, and the methyl carbon of furan appeared at 55.206 ppm, consistent with polymer formation. The solid-state ^13^C NMR spectra of V-fa polymer and PEG are displayed in Figure 3b,c.

**Figure 4:**
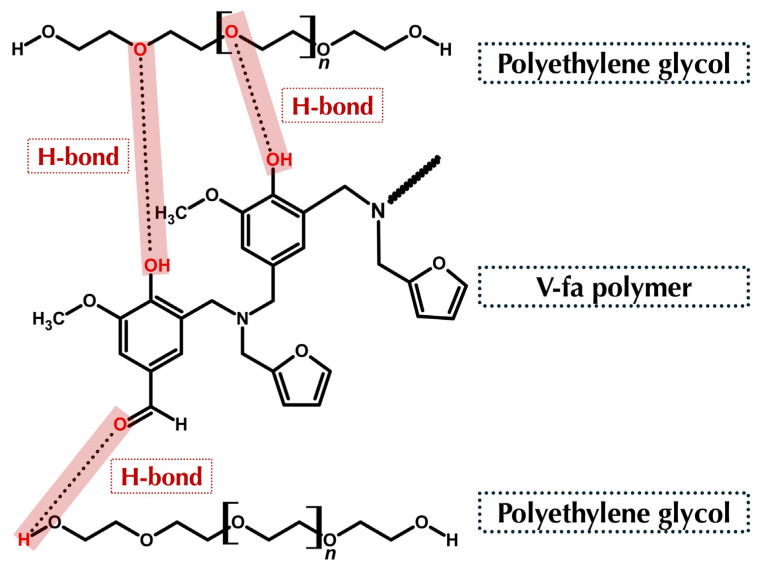
Hydrogen-bonding interactions in V-fa polymer/PEG blends.

The V-fa polymer was successfully blended with PEG to form homogeneous polymeric samples. The V-fa polymer was successfully blended with PEG to form homogeneous polymeric samples. Homogeneity of the V-fa/PEG blends, even at high PEG contents (up to 95 wt%), was ensured by thorough stirring at 130 °C for 30 min. Visual inspection, SEM images, DSC, and FTIR analyses confirmed uniform morphology, consistent thermal transitions, and hydrogen-bonding interactions without any macroscopic phase separation (Figure 2d). However, the DSC thermograms revealed a slight shift and broadening of the glass transition temperature (T_g_), indicating a partial phase separation within the blend. This behavior suggests that while V-fa and PEG are largely miscible at the molecular level due to hydrogen-bonding interactions between hydroxyl and ether groups, some microphase segregation may occur, particularly at higher PEG contents. Such partial phase separation is commonly observed in polymer blends where differences in chain flexibility and polarity influence segmental mobility and result in multiple or broadened T_g_ transitions. (Figure 2d) At lower PEG contents (50–70 wt%), the samples were homogeneous but brittle, easily fractured, and not flexible. Increasing PEG content to 80–95 wt% improved ductility and flexibility while maintaining homogeneity. FTIR analysis revealed that in blends with varying PEG contents, the intensity of CH_2_ stretching 2882–2884 cm^−1^ and C–O–C vibrations 1060–1104 cm^−1^ from PEG increased with PEG content. Moreover, the carbonyl peak of V-fa polymer at 1674 cm^−1^ shifted upon blending, indicating hydrogen-bonding interactions between the hydroxyl groups of poly(V-fa) and ether groups of PEG. These hydrogen-bonding interactions are schematically illustrated in Figure 4. This shift was more pronounced with higher PEG content or molecular weight, reflecting stronger intermolecular hydrogen bonding and explaining the enhanced flexibility of the blends. The PEG spectrum (Figure 3c) shows a prominent −O–CH_2_− signal at 70.571 ppm, which also appears in the blended system, confirming the presence of PEG chains within the polymer matrix.

Overall, the combination of FTIR (Figure 2d) and ^13^C NMR analyses (Figure 3a–c), the combination of FTIR and ^13^C NMR analyses, together with mechanical observations, demonstrates that the mechanical properties of the V-fa/PEG blends strongly depend on both PEG content and molecular weight. The increased flexibility at higher PEG loadings can be attributed to plasticization of the rigid poly(V-fa) network by PEG chains and enhanced hydrogen bonding, whereas lower PEG content results in a highly crosslinked, brittle material. These findings provide insight into tuning the mechanical behavior of bio-based benzoxazine/PEG blends through careful control of blend composition and polymer architecture [16,17].

#### 3.1.3. Water Solubility Behaviors of Polymer Blends

**Figure 5:**
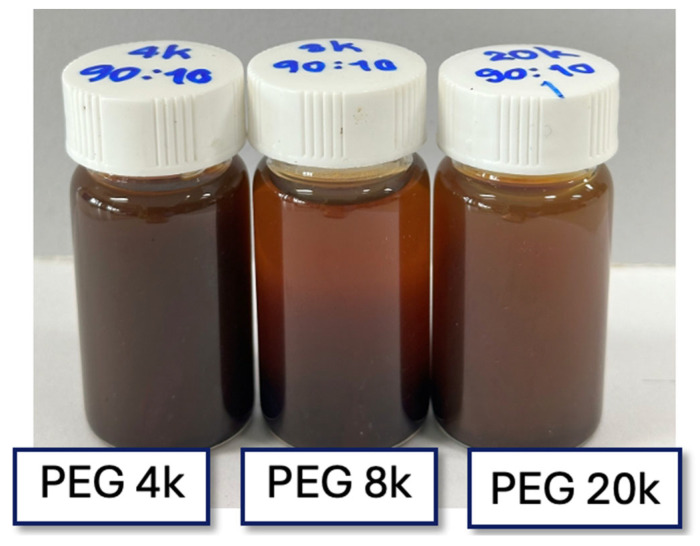
Water solubility test of V-fa polymer blends at different PEG molecular weights.

The PEG-containing blends demonstrated rapid water-induced disintegration when immersed in deionized water at room temperature. Upon contact, partial dissolution occurred within the first 5 min, and complete dissolution was observed within 30 min. The water solubility behavior of the V-fa/PEG blends at different PEG molecular weights is shown in Figure 5. This high degree of water solubility reflects the intrinsic hydrophilicity of PEG, highlighting the potential of these blends as water-degradable and environmentally responsive materials. Such behavior is particularly advantageous for applications requiring biodegradability or controlled disintegration in aqueous environments. It should be noted that, while most conventional polybenzoxazines are highly crosslinked and thus insoluble in water, the poly(V-fa) used in this study is derived from vanillin and furfurylamine, both of which contain hydrophilic functional groups (e.g., hydroxyl and furan rings). These functionalities, combined with the incomplete polymerization of some oxazine rings during curing, can result in residual hydroxyl and ether linkages that significantly enhance the polymer’s polarity and hydrogen-bonding capability toward water molecules [18,19]. Consequently, poly(V-fa) itself exhibits only partial water dispersibility or slow dissolution, particularly in finely powdered form or thin films, and is not inherently water-soluble in the sense of rapid dissolution like PEG.

To confirm this, a neat poly(V-fa) sample (without PEG) was also tested in water. The result showed that pure poly(V-fa) did not fully dissolve but became softer and slightly swollen after 2 h, demonstrating that it is only partly hydrophilic and not water-soluble. Therefore, the complete dissolution within 30 min seen in the V-fa/PEG blends arises primarily from the high PEG content and from hydrogen-bonding interactions between PEG and the residual hydroxyl/ether functionalities of poly(V-fa). These interactions effectively produce hydroxyl-rich domains and a more hydrophilic network within the blend, which facilitate water uptake, penetration, and subsequent breakdown of the polymer matrix. The hydroxyl groups associated with poly(V-fa) can form hydrogen bonds with PEG chains, improving compatibility and allowing the blends to disintegrate faster in water. This agrees with earlier studies showing that bio-based benzoxazines made from vanillin or eugenol have higher water affinity than petroleum-based ones [18,19]. In addition, PEG is well known to increase water absorption and dissolution in thermosetting polymers because of its strong hydrophilicity and flexible chains [20]. Overall, these results confirm that the fast dissolution of the V-fa/PEG system is mainly due to the combined hydrophilic effect of both the partially hydrophilic poly(V-fa) and PEG, rather than because poly(V-fa) itself is not water-soluble [15].

#### 3.1.4. Thermal Properties Characterizations of the V-fa/PEG Blends

- Thermal Behaviors of Polymer Blends

**Figure 6:**
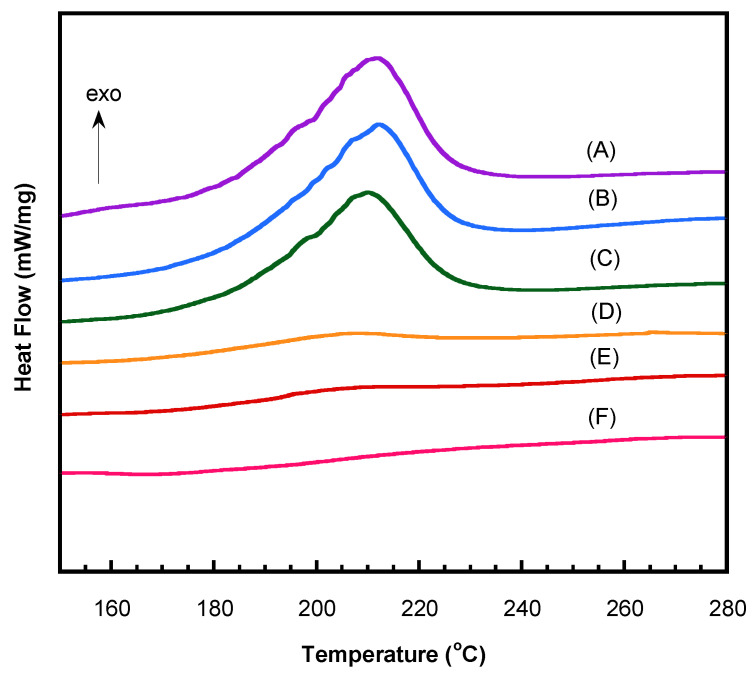
DSC thermograms to determine curing conditions of V-fa: (A) Uncured V-fa; (B) 130 °C/1 h; (C) 130 °C/1 h + 150 °C/1 h; (D) 130 °C/1 h + 150 °C/1 h + 170 °C/2 h; (E) 130 °C/1 h + 150 °C/1 h + 170 °C/2 h + 180 °C/1 h; (F) 130 °C/1 h + 150 °C/1 h + 170 °C/2 h + 180 °C/2 h.

The thermal behaviors of the bio-based V-fa benzoxazine blends with polyethylene glycol (PEG) were systematically studied using differential scanning calorimetry (DSC). The curing process of V-fa monomer was first optimized at different temperatures to promote its ring-opening polymerization into poly(V-fa). From Figure 6 and Table 1, it can be observed that the area under the melting peak decreased progressively as the curing temperature increased. The most significant reduction in peak area occurred after curing V-fa monomer at 180 °C for 2 h. Considering the percentage of conversion (% conversion) from Table 1, the uncured V-fa monomer had not undergone any polymerization. Curing at 130 °C and 150 °C resulted in only 16% and 18% conversion, respectively. The conversion increased substantially to 88% after curing at 170 °C and reached the highest value of 100% after curing at 180 °C for 2 h. These results indicate that the selected curing conditions are suitable for polymerizing the bio-based benzoxazine monomer into poly(V-fa), which is consistent with the curing conditions and results reported by Leungpuangkaew et al. (2023) and Prasomsin et al. (2019) [2,11].

In the blends with PEG, both the melting temperature (T_m_) and enthalpy (∆H) were strongly affected by PEG content and molecular weight. At lower PEG contents (50–70 wt%), the crystallinity of PEG was partially suppressed by the rigid poly(V-fa) matrix, resulting in lower ∆H values. At higher PEG contents (80–95 wt%), distinct melting transitions of PEG were observed, indicating that PEG chains could crystallize within the polymer network. The molecular weight of PEG also influenced the melting behavior: higher molecular weight PEG (20k) exhibited sharper melting peaks and higher ∆H than PEG 4k and 8k, which can be attributed to longer polymer chains and increased crystallinity.

These findings suggest that the thermal transitions of the blends are governed by a balance between the crosslink density of poly(V-fa) and the crystallization tendency of PEG. The curing process effectively converts the monomer into a fully crosslinked network, while PEG serves as a semi-crystalline soft segment. Depending on its composition and molecular weight, the thermal properties of PEG can be partially retained or suppressed. Overall, the DSC results demonstrate that controlled curing enables the successful formation of poly(V-fa) and that the thermal behavior of the blends can be tuned by adjusting PEG content and molecular weight, providing insight for designing flexible, semi-crystalline polymer systems.

**Figure 7:**
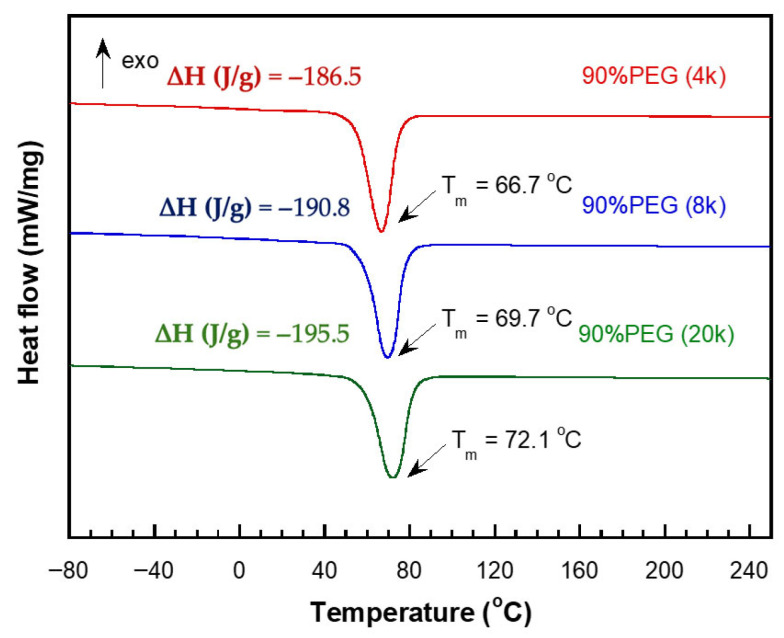
DSC thermograms (2nd heating) of blends of bio-based benzoxazine polymer (V-fa polymer) with 90 wt% PEG at different molecular weights (4000, 8000, and 20,000 Da).

Furthermore, Figure 7 presents the 2nd heating DSC analysis of blends containing 90 wt% PEG with varying molecular weights of 4000, 8000, and 20,000 Da. The T_m_ of the polymer blends increased progressively to 66.7, 69.7, and 72.1 °C with increasing PEG molecular weight. The melting enthalpy (∆H) also increased as the PEG molecular weight increased, which can be explained by the longer PEG chains enhancing intermolecular interactions within the blend, thus requiring higher energy for melting. These results are consistent with the trends reported by Pekdemir et al. (2024) and Li et al. (2020) [21,22]. This negligible variation in T_m_ can be attributed to the relatively low molecular weight of PEG (8000 Da), which limits its crystallinity and melting behavior. Additionally, the rigid poly(V-fa) network dominates the thermal behavior, partially constraining the mobility and crystallization of the PEG chains, resulting in only minor changes in T_m_ despite increasing PEG content. Overall, these results indicate that at lower molecular weight PEG, the melting transition of the blend is primarily governed by the polymer matrix rather than the PEG fraction, providing insight into the design of semi-crystalline, flexible polymer systems. [21,22].

#### 3.1.5. Determination of Thermal Stability of the V-fa/PEG Blends

**Figure 8:**
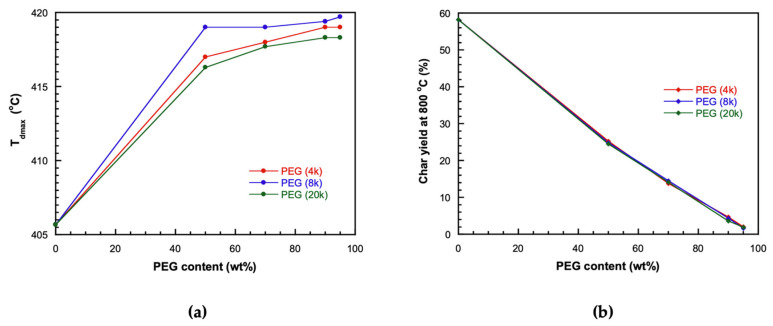
Thermal stability parameters of V-fa/PEG blends with different PEG molecular weights: (**a**) maximum decomposition temperature (T_dmax_); (**b**) char yield at 800 °C.

The thermal stability of the materials is indicated by the decomposition temperature and residual char content. After heating from room temperature to 800 °C, the remaining weight of the 8k V-fa/PEG blend is shown in Figure 8 and Table 2. The decomposition temperatures and residual char contents of blends with different PEG contents are presented in Figure 8a and Figure 8b, respectively.

**Figure 9:**
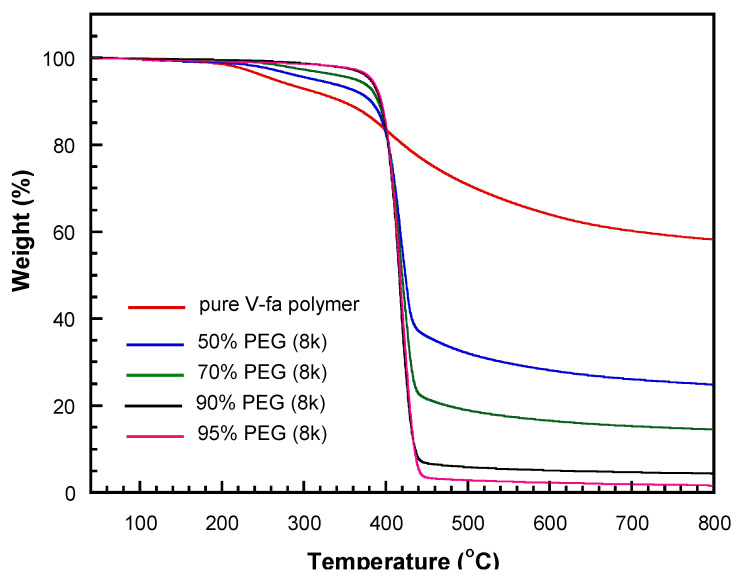
TGA thermograms of 8k V-fa/PEG blends with varying PEG contents.

The thermal stability of bio-based V-fa polybenzoxazine and its blends with polyethylene glycol (PEG) was evaluated by thermogravimetric analysis (TGA), focusing on the maximum decomposition temperature (T_dmax_). As shown in Figure 9 and Table 2, T_dmax_ was largely unaffected by PEG molecular weight when the PEG content was kept constant. For example, at 50 wt%.

For PEG, T_dmax_ values were 417.0, 419.0, and 416.3 °C for PEG 4k, 8k, and 20k, respectively, and similar trends were observed across higher PEG contents (70–95 wt%), remaining in the narrow range of 419–421 °C. At 100 wt% PEG (pure PEG polymer), T_dmax_ reached 423.0, 424.0, and 422.0 °C for PEG 4k, 8k, and 20k, respectively, showing a further slight increase with PEG content.

Similarly, when the PEG molecular weight was fixed, varying the PEG content produced only minor changes in T_dmax_. The pure V-fa polymer showed a T_dmax_ of 405.7 °C, and incorporation of PEG slightly increased T_dmax_, indicating a modest stabilizing effect from the PEG segments. These results demonstrate that the thermal degradation of V-fa/PEG blends is primarily governed by the poly(V-fa) matrix, while PEG content and chain length exert limited influence. Overall, the blends exhibit high thermal robustness across a wide range of compositions and molecular weights [23].

The residual char yield at 800 °C of the bio-based V-fa polybenzoxazine/polyethylene glycol (PEG) blends was systematically evaluated. As shown in Table 3, when PEG content was fixed, varying the molecular weight of PEG had no significant effect on the char yield. In contrast, when the molecular weight of PEG was constant, increasing PEG content led to a clear decrease in char yield. This trend is attributed to the reduced proportion of V-fa polymer, which contains thermally stable aromatic ring structures that contribute to high char formation during pyrolysis. As the PEG fraction increases, the content of these thermally robust V-fa segments decreases, resulting in lower residual char. These observations are consistent with the findings of Mora et al. (2023) [24] and highlight the key role of the polybenzoxazine network in maintaining thermal stability and char formation in the blends.

#### 3.1.6. Summary of Part I—V-fa/PEG Blends

The structural characterization of the V-fa/PEG blends using FTIR and solid-state ^13^C NMR confirmed the successful formation of the polymer blends with the desired chemical linkages. DSC analysis indicated that thermal transitions, including T_m_ and melting enthalpy (∆H), were strongly influenced by PEG content and molecular weight. Thermal stability assessed by TGA demonstrated that all blends were thermally robust, with minor variations depending on composition. Water absorption tests showed that PEG incorporation increased hydrophilicity; however, the blend at a V-fa: PEG 8k ratio of 80:20 exhibited a balanced performance, with moderate water uptake while retaining favorable thermal and mechanical properties.

Practical observations of moldability and forming quality across PEG molecular weights (4k, 8k, and 20k) revealed that PEG 8k blends exhibited the best processing performance. When evaluating V-fa: PEG 8k ratios of 50, 70, 80, 90, and 95 wt%, the 80:20 blend showed the easiest mold filling, uniform formation, smooth surfaces, and defect-free demolding. In contrast, 90 and 95 wt% blends were brittle, prone to cracking, and difficult to release from the mold, while 50 and 70 wt% blends exhibited uneven surfaces and inconsistent mold filling.

Overall, these results indicate that the V-fa: PEG 8k blend at an 80:20 ratio provides the optimal combination of thermal stability, mechanical integrity, and processability, making it the most suitable candidate for further development into polymer composites in Part II.

### 3.2. Part II—Characterizations of V-fa/PEG Polymer Composites

#### 3.2.1. Water Solubility Behaviors of Polymer Composites

From the water solubility test described in Section 3.1.3, the pure poly(V-fa) was found to be completely insoluble in water due to its highly crosslinked and rigid aromatic structure, which lacked hydrophilic functional groups capable of interacting with water molecules. The tightly packed benzoxazine network also restricted molecular motion, further preventing water diffusion and dissolution. In contrast, polyethylene glycol (PEG) is a hydrophilic polymer that readily dissolves in water because of its abundant hydroxyl (–OH) and ether (–C–O–C–) groups, which can form multiple hydrogen bonds with water molecules. When V-fa was blended with PEG to form the V-fa/PEG polymer blends, hydrogen-bonding interactions occurred between the hydroxyl groups of PEG and the phenolic or amine groups generated during the ring-opening polymerization of V-fa. These interactions led to the formation of intermolecular hydroxyl-containing networks, which improved compatibility between the two components and increased the hydrophilicity of the system.

The water solubility behavior of pure poly(V-fa), PEG, their blends, and CB-filled composites was investigated to evaluate the influence of PEG and carbon black (CB) on the hydrophilicity and stability of the materials. The results are summarized in Table S1. Pure PEG was completely soluble in water, leaving no solid residue after filtration. In contrast, pure poly(V-fa) exhibited total insolubility (100% insoluble solid) due to its highly crosslinked aromatic structure and the absence of hydrophilic functional groups. For the V-fa/PEG blend (90:10 wt%), partial dissolution occurred, leaving 21% insoluble residue, indicating the partial compatibility between the hydrophilic PEG and the rigid poly(V-fa) network. Upon incorporating 20 phr of CB into the same blend composition, the insoluble solid increased remarkably to 49%, confirming that CB significantly hindered the water penetration and dissolution process. This can be attributed to the barrier effect of the CB nanoparticles, which occupied the free volume within the polymer matrix and restricted the movement of water molecules. The CB particles were well dispersed throughout the polymer network, forming a more compact structure that limited the accessibility of water and reduced swelling during immersion.

**Figure 10:**
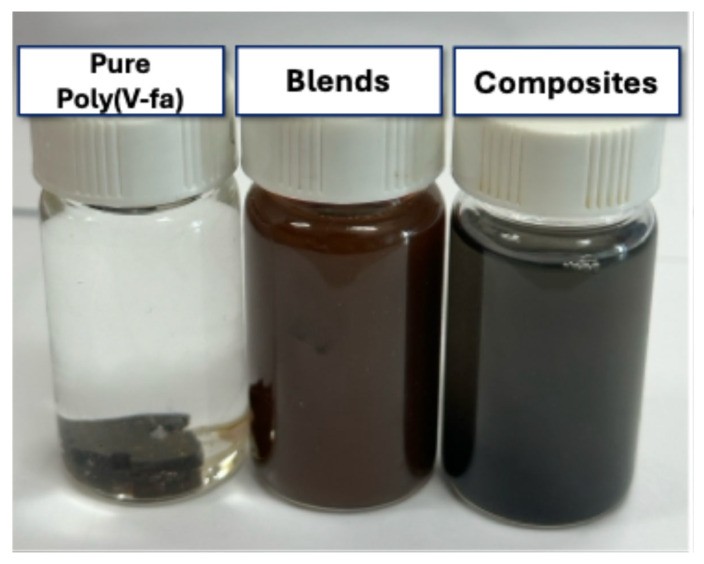
Water solubility test of pure poly(V-fa), polymer blends, and polymer composites.

As shown in Figure 10, only about 30% of the composite dissolved within the first 30 min, and approximately 50% after 2 h, while a considerable portion remained undissolved even after prolonged immersion. The presence of CB not only reduced water solubility but also enhanced the dimensional stability of the material in aqueous environments. These findings are consistent with previous studies on carbon black–reinforced polybenzoxazine systems, which reported similar improvements in water resistance [19,25].

In summary, the water solubility of the V-fa/PEG/CB composites was governed by the effects of the hydrophilic PEG phase and the barrier effect introduced by CB. The incorporation of CB substantially reduced the solubility, as reflected by the higher residual weight (49%) compared with the V-fa/PEG blend (21%), while pure poly(V-fa) remained entirely insoluble in water.

#### 3.2.2. Determination of Thermal Behaviors of the V-fa/PEG/CB Composites

The DSC results clearly demonstrated that the incorporation of CB substantially influences the thermal transitions of V-fa/PEG composites. The melting temperature (T_m_) of neat PEG-rich blends (70–90 wt% PEG without CB) was consistently observed at ≈70 °C, in good agreement with the crystalline melting point of semi-crystalline PEG phases. Upon addition of CB, small increases in T_m_ were detected. For example, the T_m_ values at CB content ranging from 2.5 to 20 phr shifted from 70 to 72 °C, suggesting that CB particles can act as effective heterogeneous nucleating agents. The available filler surface could promote the growth of more ordered PEG crystallites, thereby elevating the melting point slightly compared to the neat blend. Similar nucleation-induced T_m_ enhancements have been reported in PEG/carbon nanotube and PEG/graphene oxide systems, where filler surfaces facilitate lamellar thickening or selection of more perfect crystalline domains [26,27], as summarized in Table 4.

Despite the slight increase of T_m_, the associated melting enthalpy (∆H_m_) decreased progressively with CB content (from 207 J/g at 80:20 blend without CB to only 156 J/g at 20 phr CB), as shown in Table 4. This decline in ∆H_m_ indicates a reduction in the overall crystallinity of the PEG phase. The presence of non-crystallizable filler particles effectively reduces the available free volume for PEG chain alignment and increases geometric constraints at the polymer–filler interface. Consequently, fewer lamellae are formed, and imperfect crystallization dominates, lowering the overall enthalpic contribution of melting. This trend is consistent with the dilution and immobilization effects widely observed for carbon-filled semi-crystalline polymers [27,28,29].

At the highest CB loading (20 phr), T_m_ exhibited an anomalous increase to 83 °C, while ∆H_m_ dropped sharply. Such divergence may arise from several factors: selective nucleation of highly perfect but sparse crystalline domains at the filler surface, phase separation between PEG and poly(V-fa) promoted by high filler loading, or experimental artifacts such as thermal contact heterogeneity in samples with uneven filler dispersion. Given the magnitude of the T_m_ shift.

The crystallization temperature (T_c_) of PEG phases was less sensitive to CB addition, generally remaining in the 32–35 °C range across most filler loadings. However, at 20 phr CB, T_c_ dropped significantly to 26 °C, consistent with the crystallization hindrance. High filler concentrations can impede chain diffusion during cooling, increase interfacial viscosity, and cause aggregation that obstructs nucleation pathways. The associated decrease in crystallization enthalpy (∆H_c_) further supports the view that PEG crystallization becomes less efficient under these conditions. Comparable reductions in T_c_ at elevated CB or carbon nanotube loadings have been reported in PEG/polyurethane and PEG/epoxy matrices [30,31] highlighting the universal hindrance effect of excessive filler contents on polymer crystallization.

In addition, the glass transition temperature (T_g_) of the composites exhibited a gradual upshift with increasing CB concentration, reflecting enhanced segmental restriction at the polymer–filler interface. This effect is attributed to strong interfacial interactions, likely hydrogen bonding between PEG ether groups and surface functionalities of CB or physical adsorption of PEG chains onto the filler surface. The restricted mobility increases the effective stiffness of the amorphous phase and translates into improved dimensional stability at elevated temperatures. Similar T_g_ increases due to polymer–filler interfacial immobilization has been widely reported in CB-reinforced elastomers and thermoplastic composites [9,10]. The corresponding T_g_ data are provided in Table 4.

Conclusively, the DSC results revealed a dual role of CB in the V-fa/PEG system. While CB could promote nucleation and slightly elevate T_m_ at low contents, its dominant effect was to suppress crystallinity, reduce enthalpy, and restrict molecular mobility at higher loadings. These observations are in strong agreement with prior studies of carbonaceous nanofillers in semi-crystalline matrices, and they highlight the importance of optimizing filler loading to balance crystallinity, thermal stability, and mechanical reinforcement.

#### 3.2.3. Determination of Thermal Stability of the V-fa/PEG/CB Composites

Thermal degradation of polymer composites of the materials is indicated by the decomposition temperature and residual char content. After heating from room temperature to 800 °C, the remaining weight of the 8k V-fa/PEG/CB composites is shown in Table 5.

The thermal stability of V-fa/PEG composites with varying PEG content (80 and 90 wt%) and CB loadings (0–20 phr) revealed the maximum decomposition temperature (T_dmax_) and char yield at 800 °C, which are summarized in Table 5. For the 80 wt% PEG composites, T_dmax_ remained nearly constant in the range from 416 to 419 °C regardless of CB content, indicating that the addition of CB does not significantly affect the primary thermal degradation mechanism of the polymer matrix and behaves as a thermally inert filler. Notably, the char yield at 800 °C increased progressively with higher CB loadings, rising from 10.78% for the neat blend to 11.23%, 17.54%, 22.82%, and 23.31% for 5, 10, 15, and 20 phr CB, respectively. A similar trend was observed in the 90 wt% PEG composites, where T_dmax_ was only slightly affected, varying from 419 °C for the neat polymer to 418 °C, 417 °C, and 417 °C for 5, 10, and 20 phr of CB, respectively, while the char yield increased from 10.78% to 11.37%, 15.45%, and 21.35%. The observed increase in char formation with higher CB content was attributed to the intrinsic thermal stability and non-volatile carbonaceous nature of CB, promoting the formation of a continuous carbonaceous network on the material surface. Additionally, CB acted as a physical barrier that suppressed heat [32] and gas transfer, effectively retarding thermal decomposition and improving fire resistance. Despite these increases in char yield, the T_dmax_ of all samples remained essentially unchanged, demonstrating that CB enhances thermal stability without altering the fundamental degradation behaviors of the polymer matrix. Conclusively, the TGA results highlight the important role of CB as a reinforcement additive that improves char yield and fire resistance while maintaining the polymer’s inherent thermal degradation profile, and this trend is consistent across both 80 wt% and 90 wt% V-fa/PEG composites.

#### 3.2.4. Mechanical Properties of V-fa/PEG/CB Composites

**Figure 11:**
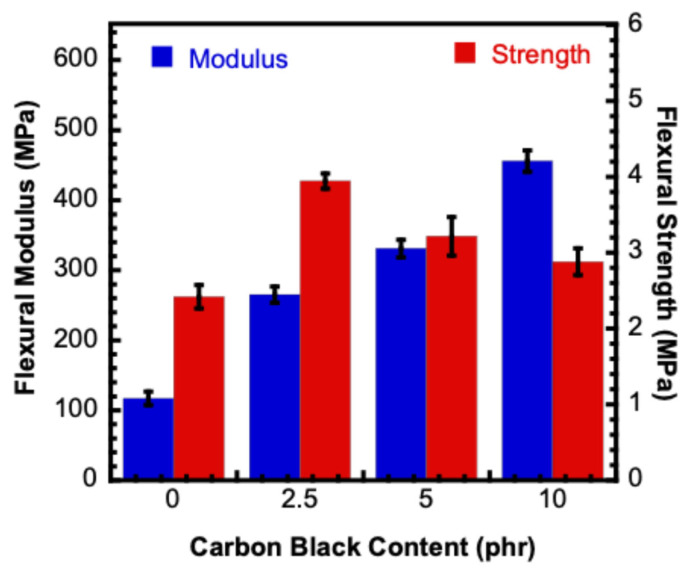
Flexural modulus and flexural strength of V-fa/PEG/CB composites with varying CB contents.

The flexural properties of the V-fa/PEG composites reinforced with CB contents (0–10 phr) were evaluated under three-point bending according to ASTM D790, and the results are summarized in Figure 11. The neat composite (0 phr) exhibited a flexural modulus of 117.80 ± 9.50 MPa, with a corresponding yield strength of 1.87 ± 0.01 MPa and flexural strength of 2.42 ± 0.15 MPa.

Upon the incorporation of 2.5 phr of CB, the flexural modulus increased markedly to 295.12 ± 29.83 MPa, while the maximum stress improved significantly to 3.94 ± 0.10 MPa, despite a slight reduction in yield strength to 1.25 ± 0.07 MPa. At 5 phr CB, a further increase in modulus was observed, 331.04 ± 12.38 MPa, although the maximum stress decreased to 3.22 ± 0.25 MPa, with the yield strength maintained at 1.24 ± 0.12 MPa. The composites with 10 phr CB demonstrated the highest stiffness, reaching a flexural modulus of 455.76 ± 5.10 MPa, but both yield strength (1.20 ± 0.09 MPa) and maximum stress (2.88 ± 0.17 MPa) declined compared with the 2.5 and 5 phr composites [33].

These results clearly demonstrate that the addition of CB significantly enhances the stiffness of the V-fa/PEG composites, with the modulus exhibiting a steady increase as the filler content rises. However, the strength behaviors followed a different trend, where the maximum flexural stress peaked at 2.5 phr and gradually declined at higher filler loadings. This indicates that at low filler content, well-dispersed CB particles effectively restrict the polymer chain mobility and enable efficient stress transfer across the filler–matrix interface, leading to enhanced strength. At higher loadings, however, the tendency of CB to agglomerate likely introduced stress concentration sites, thereby reducing the flexural strength despite the continued rise in modulus.

Such behavior is consistent with previous studies on CB-reinforced polymer composites, where filler dispersion and interfacial adhesion are considered critical factors in governing mechanical performance [32]. Overall, the findings confirm that CB acts as an efficient reinforcing filler, substantially improving flexural stiffness and providing strength enhancement at moderate loadings.

Following the flexural tests, the fractured specimens were collected, and their fracture surfaces were further examined by scanning electron microscopy (SEM) to investigate the failure mechanisms and to correlate CB dispersion and agglomeration with the observed mechanical properties [34,35].

#### 3.2.5. Morphology of Fracture Surface of V-fa/PEG Blends

**Figure 12:**
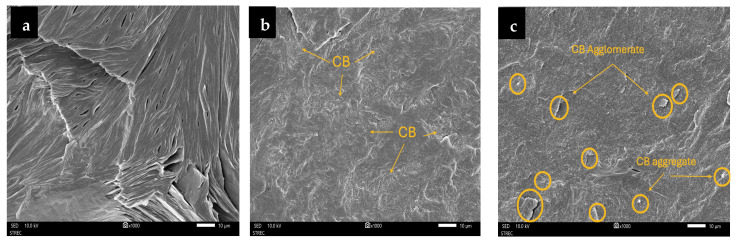
SEM micrographs of the fracture surfaces of V-fa/PEG composites after flexural testing, showing the effect of CB loading: (**a**) 0 phr; (**b**) 2.5 phr; (**c**) 5 phr (at ×1000 magnification).

SEM images of the V-fa/PEG composites shown in Figure 12 revealed distinct changes in fracture morphology depending on the CB content. The neat composite (0 phr) exhibited a rough and irregular fracture surface with numerous micro-voids and pronounced ridges, indicative of ductile fracture behavior where polymer chains underwent significant deformation before failure. After the incorporation of 2.5 phr CB, the fracture surfaces remained relatively rough, without noticeable agglomeration, suggesting good dispersion of CB particles. This dispersion contributed to the observed increase in maximum flexural stress (3.94 ± 0.10 MPa) and marked increase in modulus (295.12 ± 29.83 MPa), while yield strength slightly decreased (1.25 ± 0.07 MPa). At 5 phr, small CB aggregates began to appear, reflected by localized clustering on the fracture surface, correlating with a moderate reduction in maximum stress (3.22 ± 0.25 MPa) despite further increase in stiffness (331.04 ± 12.38 MPa) and stable yield strength (1.24 ± 0.12 MPa). At the highest CB loading (10 phr), SEM revealed larger aggregates and smoother, flattened regions, indicative of brittle fracture. The restricted chain mobility and extensive polymer–filler interfacial regions reduced plastic deformation, consistent with the decrease in yield strength (1.20 ± 0.09 MPa) and maximum stress (2.88 ± 0.17 MPa), despite the highest modulus (455.76 ± 5.10 MPa).

Conclusively, the SEM results confirm that CB effectively reinforces the V-fa/PEG composites at low to moderate loadings by enhancing stiffness while maintaining ductile characteristics. However, at higher loadings (5–10 phr), filler agglomeration dominates, leading to stress concentration, reduced flexural strength, and a transition toward brittle fracture, consistent with the UTM results [36,37].

## 4. Conclusions

In summary, this study successfully improved the brittleness of bio-based V-fa benzoxazine by blending it with PEG and reinforcing it with carbon black (CB) to achieve enhanced flexibility, processability, and overall performance. The V-fa/PEG 8k blend at 80:20 wt% was identified as the optimal matrix, providing good processability and mechanical stability. The incorporation of CB significantly influenced the composites’ properties. Thermal analyses (DSC, TGA) revealed slight increases in T_m_ and T_g_, high decomposition temperatures (~416 °C), and enhanced char yield up to 28% at 20 phr CB. Mechanical testing using UTM under three-point bending demonstrated that the flexural modulus steadily increased with CB content, reaching a maximum of 455.76 ± 5.10 MPa at 10 phr. The maximum flexural stress, however, peaked at 2.5 phr CB (3.94 ± 0.10 MPa) and gradually decreased at higher loadings due to filler agglomeration, while yield strength slightly decreased with increasing CB content. These results indicate that low CB content enables efficient stress transfer and ductile behavior, whereas high CB content enhances stiffness but reduces toughness. SEM analysis confirmed this trend, showing ductile fracture at low CB loading and brittle fracture at higher contents. Overall, the results demonstrate that low-level CB addition effectively reinforces the V-fa/PEG system, improving both mechanical performance and thermal stability, and offering a sustainable route to high-performance composites. Moreover, the unique combination of PEG-induced flexibility and CB reinforcement suggests strong potential for future development in smart materials and shape memory polymers, enabling multifunctional applications such as environmentally responsive, self-healing, or shape-recoverable systems.

## Figures and Tables

**Table 1:** Percentage of polymerization (% conversion) of V-fa monomer at different curing temperatures.

Curing Conditions of V-fa Monomer at Different Temperatures	Curing Enthalpy (∆H, J/g)	Degree of Polymerization (% Conversion)
Uncured V-fa monomer	28	0
130 °C /1 h	24	16
130 °C /1 h + 150 °C/1 h	23	18
130 °C /1 h + 150 °C/1 h + 170 °C/2 h	3	88
130 °C/1 h + 150 °C/1 h + 170 °C/2 h + 180 °C/1 h	2	94
130 °C/1 h + 150 °C/1 h + 170 °C/2 h + 180 °C/2 h	0	100

**Table 2:** Maximum decomposition temperature (T_dmax_) of the blends between bio-based V-fa polybenzoxazine and polyethylene glycol (PEG) at different contents.

PEG Content (wt%)	Maximum Decomposition Temperature (T_dmax_) (°C)		
	PEG (4k)	PEG (8k)	PEG (20k)
pure V-fa polymer	405.7	405.7	405.7
50	417.0	419.0	416.3
70	420.0	421.0	420.0
80	420.0	419.0	419.0
90	420.0	421.0	421.0
95	419.0	419.7	418.3
pure PEG polymer	423.0	424.0	422.0

**Table 3:** Char yield at 800 °C of bio-based polybenzoxazine/PEG blends with varying PEG contents.

PEG Contents (wt%)	Char Yield 800 °C (%)		
	PEG (4k)	PEG (8k)	PEG (4k)
pure V-fa polymer	58	58	58
50	25	25	25
70	15	14	13
80	9	11	11
90	6	5	4
95	2	2	2
pure PEG polymer	0	0	0

**Table 4:** T_m_, ∆H_m_, T_c_, ∆H_c_, and T_g_ (°C) of V-fa polymer/PEG blends with varying PEG contents.

8k PEG Content (wt%)	CB Content (phr)	T_m_ (°C)	∆H_m_ (J/g)	T_c_ (°C)	∆H_c_ (J/g)	T_g_ (°C)
70	0	70	192	34	146	–53
80	0	70	207	32	166	–53
80	5	71	188	34	161	–53
80	10	72	173	35	148	–52
80	20	83	156	26	140	–48
90	0	70	209	32	184	–52

**Table 5:** Maximum decomposition temperature and char yield at 800 °C of bio-based V-fa polybenzoxazine/PEG/CB composites with varying CB contents.

PEG Content (wt%)	70					80					90				
CB Content (phr)	0	5	10	15	20	0	5	10	15	20	0	5	10	15	20
(T_dmax_) (°C)	421	420	419	416	414	419	416	416	416	416	419	418	417	417	417
Char yield 800 °C (%)	14	17	22	26	28	10	11	18	23	23	5	11	15	19	21

## Data Availability

The original contributions presented in the study are included in the article; further inquiries can be directed to the corresponding author.

## Supplementary Materials

The following supporting information can be downloaded at https://www.mdpi.com/article/10.3390/polym17202776/s1: Figure S1: FTIR spectra of polymers blends (70 wt% PEG) with different molecular weights (4k, 8k, and 20k).; Figure S2: FTIR spectra of V-fa/PEG (80 wt% PEG, M_n_ = 8k) composites with varying carbon black (CB) contents from 0 to 20 phr.; Figure S3: Flexural stress–strain curves of V-fa/PEG (80 wt% PEG, M_n_ = 8k) composites with varying carbon black (CB) contents from 0 to 20 phr; Figure S4: SEM micrographs of V-fa/PEG (80 wt% PEG, M_n_ = 8k) composites with different carbon black (CB) contents: (a) 0 phr, (b) 2.5 phr, (c) 5 phr, and (d) 10 phr, observed at magnifications of 30×, 100×, and 1000×; Table S1: Percentage of polymerization (% conversion) of V-fa monomer at different curing temperatures; Table S2: Forming results of bio-based polybenzoxazine (V-fa polymer) blended with poly(ethylene glycol) (PEG) having a molecular weight (M_n_) of 8000 Da at various ratios; Table S3: Forming results of bio-based polybenzoxazine (V-fa polymer) blended with poly(ethylene glycol) (PEG) having a molecular weight (M_n_) of 20,000 Da at various ratios.; Table S4: Water solubility test of pure poly(V-fa), polymer blends and polymer composites contents.
