# Supplementary material for: Development of Bio-Based Benzoxazine V-fa/PEG/Carbon Black Composites: Thermal and Mechanical Properties
Source: Polymers (Basel). 2025 Oct 16;17(20):2776. doi: 10.3390/polym17202776 (PMC12567345; doi:10.3390/polym17202776)
Supplement: Supplementary file 1 [file polymers-17-02776-s001.zip › polymers-3915195-supplementary.pdf]

Supplementary file

Development of Bio-Based Benzoxazine V-fa/PEG/Carbon Black Composites: Thermal and Mechanical Properties

Nattapon Chaiwichian [1], Chaitawat Saelee [1], Kamontip Kuttiyawong [1], Sarawut Rimdusit [2], Kasinee Hemvichian [3], Pattra Lertsarawut [3], and Sunan Tiptipakorn [1,*]

[1] Department of Physical and Material Sciences, Faculty of Liberal Arts and Science, Kasetsart University, Nakhon Pathom 73140, Thailand; nattapon.chaiw@ku.th (N.C.); chaitawat.sa@ku.th (C.S); faaskmt@ku.ac.th (K.K)
[2] Center of Excellence in Polymeric Materials for Medical Practice Devices, Department of Chemical Engineering, Faculty of Engineering, Chulalongkorn University, Bangkok 10330, Thailand; sarwut.r@chula.ac.th
[3] Thailand Institute of Nuclear Technology (Public Organization), Ongkarak District, Nakornnayok 26120, Thailand; kasinee@tint.or.th (K.H.); pattra@tint.or.th (P.L.)
* Correspondence: faassntk@ku.ac.th; Tel.: +66-34-281105

Table of contents

Figure S1: FTIR spectra of polymers blends (70 wt% PEG) with different molecular weights (4k, 8k, and 20k).

Figure S2: FTIR spectra of V-fa/PEG (80 wt% PEG, Mn = 8k) composites with varying carbon black (CB) contents from 0 to 20 phr.

Figure S3: Flexural stress–strain curves of V-fa/PEG (80 wt% PEG, Mn = 8k) composites with varying carbon black (CB) contents from 0 to 20 phr.

Figure S4: SEM micrographs of V-fa/PEG (80 wt% PEG, Mn = 8k) composites with different carbon black (CB) contents: (a) 0 phr, (b) 2.5 phr, (c) 5 phr, and (d) 10 phr, observed at magnifications of 30×, 100×, and 1000×.

Table S1. Percentage of polymerization (% conversion) of V-fa monomer at different curing temperatures.

PEG content (wt%)	Forming result	Observed characteristics	Flexibility (bending ability)
0 (pure V-fa polymer)	Successfully formed	Rigid, brittle	Not possible
50	Successfully formed	Rigid, brittle	Not possible
70	Successfully formed	Rigid, brittle	Not possible
80	Successfully formed	Flexible	possible
90	Successfully formed	Flexible	possible
95	Successfully formed	Flexible	possible

Table S2. Forming results of bio-based polybenzoxazine (V-fa polymer) blended with poly(ethylene glycol) (PEG) having a molecular weight (M_n) of 8000 Da at various ratios.

PEG content (wt%)	Forming result	Observed characteristics	Flexibility (bending ability)
0 (pure V-fa polymer)	Successfully formed	Rigid, brittle	Not possible
50	Successfully formed	Rigid, brittle	Not possible
70	Successfully formed	Rigid, brittle	Not possible
80	Successfully formed	Flexible	possible
90	Successfully formed	Flexible	possible
95	Successfully formed	Flexible	possible

Table S3. Forming results of bio-based polybenzoxazine (V-fa polymer) blended with poly(ethylene glycol) (PEG) having a molecular weight (M_n) of 20000 Da at various ratios.

PEG content (wt%)	Forming result	Observed characteristics	Flexibility (bending ability)
0 (pure V-fa polymer)	Successfully formed	Rigid, brittle	Not possible
50	Successfully formed	Rigid, brittle	Not possible
70	Successfully formed	Rigid, brittle	Not possible
80	Successfully formed	Flexible	possible
90	Successfully formed	Flexible	possible
95	Successfully formed	Flexible	possible

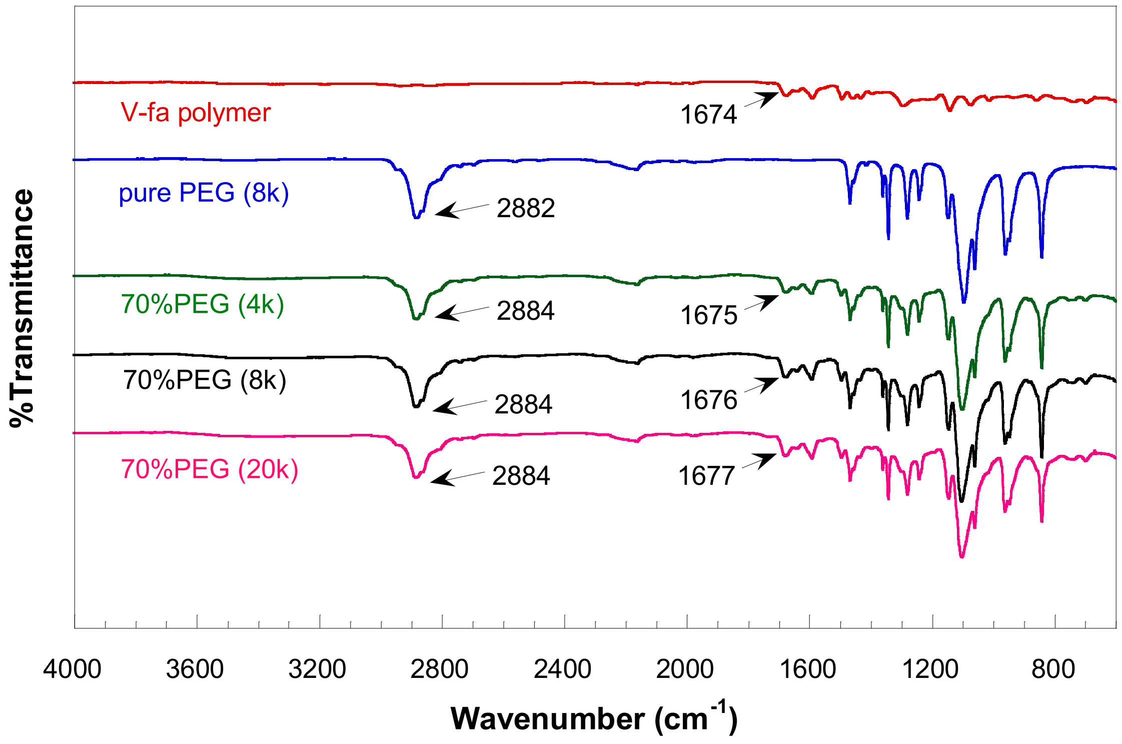

Figure S1. FTIR spectra of polymers blends (70 wt% PEG) with different molecular weights (4000, 8000, and 20000 Da).

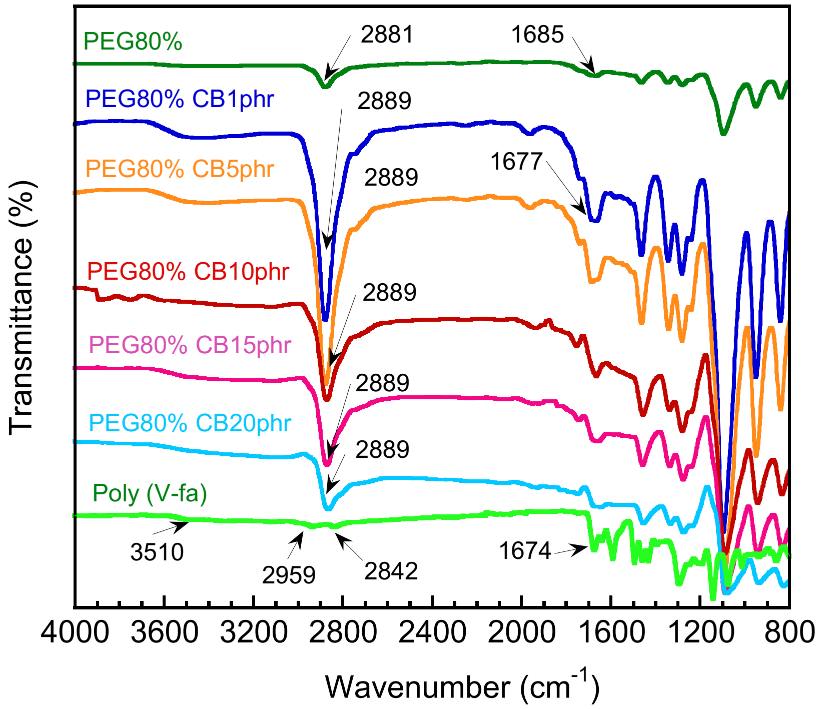

Figure S2. FTIR spectra of V-fa/PEG (80 wt% PEG, M_n = 8k) composites with varying carbon black (CB) contents from 0 to 20 phr.

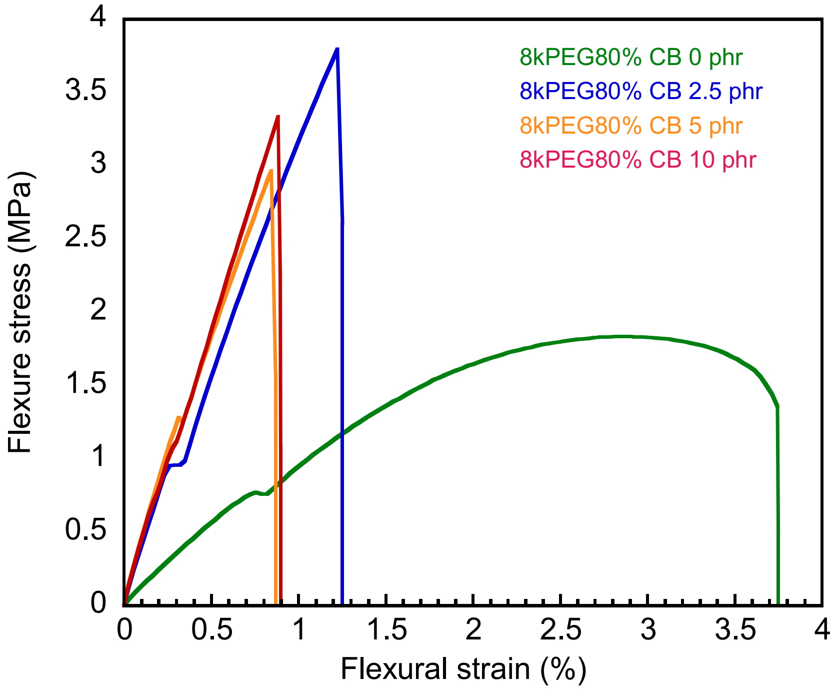

Figure S3. Flexural stress–strain curves of V-fa/PEG (80 wt% PEG, M_n = 8k) composites with varying carbon black (CB) contents from 0 to 20 phr.

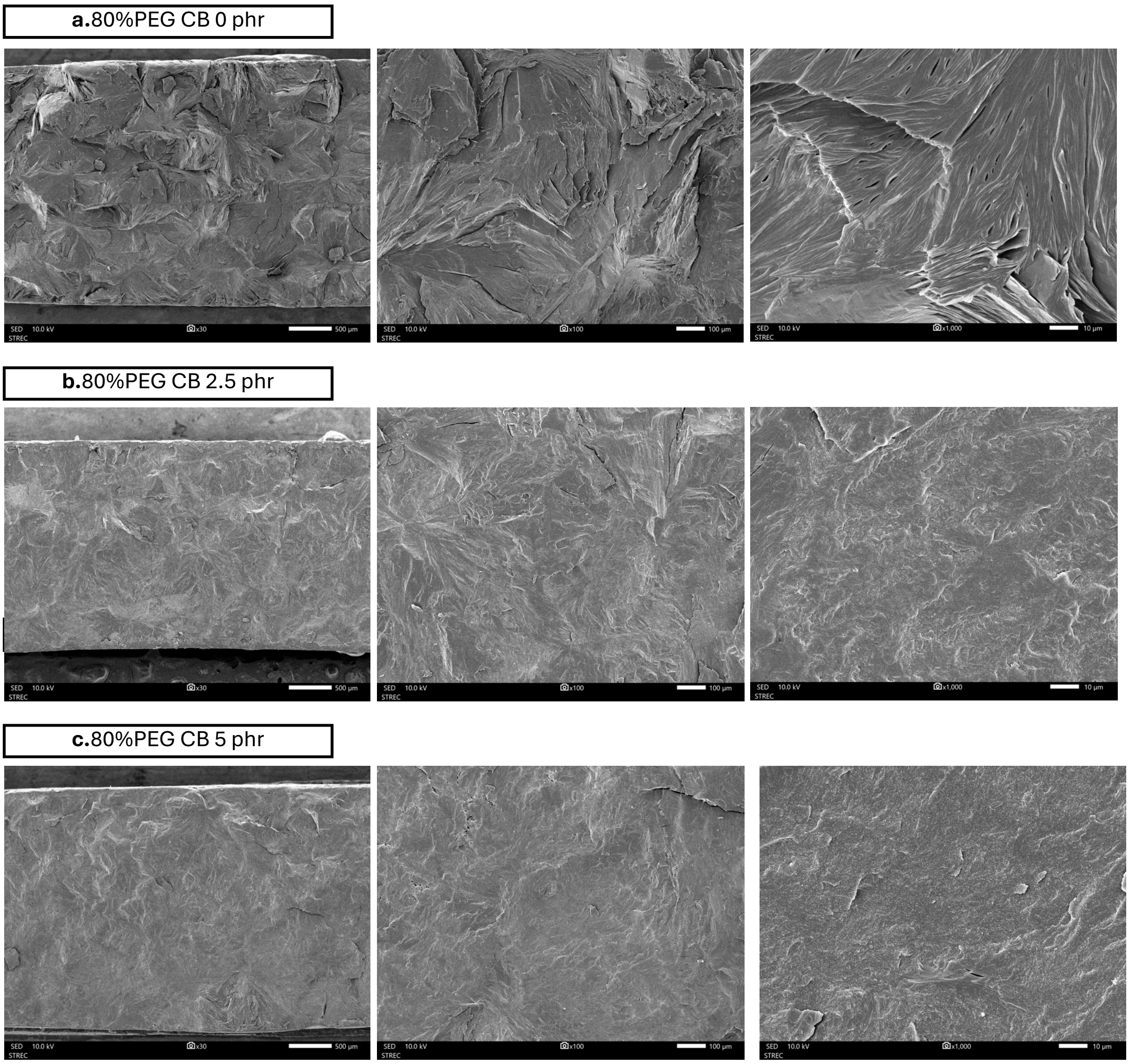

Figure S4. SEM micrographs of V-fa/PEG (80 wt% PEG, M_n = 8k) composites with different carbon black (CB) contents: (a) 0 phr, (b) 2.5 phr, and (c) 5 phr, observed at magnifications of 30×, 100×, and 1000×.

Table S4. Water solubility test of pure poly(V-fa), polymer blends and polymer composites contents.

PEG Content (wt%)	Insoluble Solid Left Behind on the Filter Paper (%)
100:0 (Pure PEG)	0
90:10 Blends w/o CB	21
90:10 Composites w/n CB 20 phr (16.7 %)	49
0:100 (Pure V-fa)	100

* Whatman filter paper No.1
